# Inhibiting Effects of Antibiotic-Loaded Porous Gelatin-Hydroxyapatite Microspheres on *Staphylococcus aureus*

**DOI:** 10.3390/pharmaceutics17121598

**Published:** 2025-12-11

**Authors:** Meng-Ying Wu, Chao-Chun Yen, Ming-Jia Wang, I-Fang Kao, Shiow-Kang Yen

**Affiliations:** 1 Department of Materials Science and Engineering, National Chung Hsing University, Taichung 402, Taiwan; slimu16882013@gmail.com (M.-Y.W.);; 2 Department of Orthopedics, Taichung Armed Forces General Hospital, Taichung 404, Taiwan; 3 Department of Orthopedics, National Defense Medical University, Taipei 114, Taiwan; 4 Department of Healthcare Administration, Central Taiwan University of Science and Technology, Taichung 406, Taiwan; 5 Department of Gerontological Health Care, Central Taiwan University of Science and Technology, Taichung 406, Taiwan; 6 Department of Dental Technology and Material Science, Central Taiwan University of Science and Technology, Taichung 406, Taiwan

**Keywords:** hydroxyapatite, gelatin, microsphere, drug load and release, antibacterial, cell culture

## Abstract

**Background/Objectives:** Due to their biocompatibility and bone-like composition, calcium phosphate materials—especially hydroxyapatite (HAp)—have emerged as promising carriers for localized antibiotic delivery in bone regeneration. Here, we developed Hap-based composite microspheres using a simple wet-chemical method and incorporated multiple antibiotics to evaluate their release profiles and antibacterial potential for treating bone infections. **Methods:** In this study, uniform and porous composite microspheres composed of Hap and gelatin were synthesized via a simple wet-chemical method using a mixed calcium phosphate–gelatin solution. **Results:** The resulting gelatin–Hap microspheres (G-HAM) were systematically characterized to verify their crystalline structure, morphology, composition, and thermal stability. G-HAM exhibited a highly porous structure, making them well-suited for use as drug carriers. Four clinically relevant antibiotics—gentamicin, vancomycin, teicoplanin, and zyvox—were incorporated into the microspheres and evaluated for their release behavior and antibacterial performance against *Staphylococcus aureus*. The release profiles revealed an initial burst release within the first hour that exceeded the minimum inhibitory concentrations of all tested antibiotics, followed by a sustained release phase. Antibiotics containing carboxylic groups, such as vancomycin and teicoplanin, demonstrated stronger interactions with Hap, resulting in a more prolonged release. Antibacterial testing confirmed that the released antibiotics maintained their chemical stability and bioactivity. Furthermore, the combination of bioactive Hap and peptide-rich gelatin promoted osteoblast-like cell adhesion and proliferation, while cytotoxicity assays verified excellent biocompatibility. **Conclusions:** Overall, these G-HAM provide a promising platform that integrates controlled antibiotic release with osteoconductive potential for bone infection treatment and tissue regeneration.

## 1. Introduction

Mineralized tissues such as bone and dentin are frequently affected by disease or trauma, necessitating effective regenerative strategies. Calcium phosphate (CaP) bio-ceramics, due to their compositional similarity to bone, excellent biocompatibility, and osteo-conductive/osteo-inductive properties, have emerged as promising candidates for drug and ion delivery systems to support craniofacial tissue regeneration [1]. The family of CaP biomaterials exhibit excellent biocompatibility and osteo-conductivity, and in some cases osteo-inductivity, while their inherent brittleness and poor mechanical strength limit their use in load-bearing applications. To overcome this, CaP is often coated onto metallic implants like titanium alloys, combining mechanical strength with bioactivity and enhancing stability, osseointegration, and clinical performance in orthopedic and dental use [2,3]. Among CaP ceramics, hydroxyapatite (HAp) stands out as the primary focus of research.

HAp, due to its close chemical composition and crystal structure to the inorganic components of human bone and teeth, has been widely applied in bone tissue engineering. This similarity endows HAp with several desirable properties, including good biocompatibility, osteo-conductivity, and a certain degree of biodegradability, as well as potential applications as a drug carrier and in promoting tissue regeneration. Various methods have been developed to synthesize HAp, including the wet chemical method [4,5], microwave-assisted hydrothermal [6,7,8], chemical precipitation [9], sol-gel [10,11,12], and micro/emulsion techniques [13,14].

In natural bone, the extracellular matrix is primarily composed of type I collagen and HAp microcrystals, forming a composite structure that combines hardness with toughness [15]. Owing to its chemical similarity to the inorganic phase of bone, HAp exhibits excellent biocompatibility and osteo-conductivity, making it widely applied in bone tissue engineering [16]. However, pure HAp suffers from brittleness and insufficient mechanical strength, which limits its use in load-bearing applications [17,18]. To overcome these drawbacks, many studies have focused on combining HAp with natural polymers—such as collagen [19,20], chitosan [16,21], alginate [22,23,24], gelatin [25,26], and silk fibroin [27,28]—to enhance its mechanical properties and biological performance, better mimicking the structure and function of native bone.

Vancomycin, gentamicin, teicoplanin, and zyvox are commonly used antibiotics for treating severe infections caused by Gram-positive bacteria, including methicillin-resistant *Staphylococcus aureus* (MRSA) and vancomycin-resistant *Enterococcus* (VRE) [29,30,31]. Vancomycin and teicoplanin, both glycopeptide antibiotics, are widely applied in the management of osteomyelitis and soft tissue infections [32,33,34], while gentamicin, an aminoglycoside antibiotic, exhibits broad-spectrum antibacterial activity [29,35]. Zyvox, an oxazolidinone antibiotic, is a synthetic agent effective against multidrug-resistant Gram-positive pathogens [30,36].

In recent years, various drug delivery systems (DDSs) have been developed to improve the therapeutic efficacy and local bioavailability of these antibiotics [37]. For example, they have been incorporated into nanoparticles, hydrogels, nanofibers, and bone cement composites, aiming to achieve localized and sustained release while reducing systemic toxicity [33,38,39]. Such DDS-based strategies can enhance antibacterial efficacy and promote tissue regeneration at the infection site. However, these systems still face challenges such as initial burst release, rapid carrier degradation, limited large-scale production feasibility, and potential local cytotoxicity [36,40].

To address these challenges, this study aimed to develop a HAp-based composite material as an antibiotic carrier. The composite microspheres were synthesized via a simple, low-cost wet-chemical method without the need for organic solvents or cross-linkers and systematically characterized by x-ray diffraction (XRD), Fourier-transform infrared spectroscopy (FT-IR), scanning electron microscope (SEM), inductively coupled plasma mass spectrometry (ICP-MS), and thermal analyses. Widely used antibiotics, including gentamicin, vancomycin, teicoplanin, and zyvox, were incorporated to investigate their release behavior and antibacterial potential. This approach seeks to provide a promising platform for bone infection treatment.

## 2. Materials and Methods

### 2.1. Material Preparation and Characterization

#### 2.1.1. Synthesis of Porous Gelatin-HAp Composite Microspheres (G-HAM)

Porous G-HAM were synthesized via a wet-chemical precipitation method. Ca(NO_3_)_2_·4H_2_O (SHOWA, Tokyo, Japan) and NH_4_H_2_PO_4_ (SHOWA, Tokyo, Japan) were dissolved separately in deionized water and then added into a 4 wt.% gelatin (Sigma-Aldrich, St. Louis, MO, USA) solution, prepared by dissolving gelatin in deionized water at 65 °C, under continuous stirring, maintaining a Ca/P ratio of 1.67. The mixture was kept at a controlled temperature until precipitation formed, followed by centrifugation, washing, filtration, and drying to yield white microsphere powders. According to previous experience [41], this synthesis procedure was based on established methods, and the obtained powders were compared with reagent-grade HAp (Sigma-Aldrich, St. Louis, MO, USA).

#### 2.1.2. Scanning Electron Microscopy (SEM)

The morphology of the microspheres was examined using scanning electron microscopy (SEM, JSM-5400 and JSM-6700F, JEOL, Tokyo, Japan). For sample preparation, the powders were dispersed on conductive carbon tape attached to aluminum stubs and sputter-coated with gold prior to imaging.

#### 2.1.3. X-Ray Diffraction (XRD)

Phase identification was performed using X-ray diffraction (XRD, MXP-III, Bruker, Billerica, MA, USA) with a CuKα source (*λ* = 1.542 Å), operating at 40 kV and 30 mA. Data were collected in the range of 2θ from 10° to 70° at a scanning rate of 2°/min. The crystallite size along the (002) plane was estimated by the Scherrer equation:

*d* = *k λ*/(*b* cos*θ*) (1)

where *d* represents the crystallite diameter, *k* is the shape factor (0.9), *λ* denotes the X-ray wavelength, *b* is the full width at half maximum (FWHM) of the diffraction peak, and *θ* is the Bragg angle corresponding to the (002) plane.

#### 2.1.4. Transmission Electron Microscopy (TEM)

The internal structure of microspheres was observed using transmission electron microscopy (TEM, JEM-200CX and JEM-2010, JEOL, Tokyo, Japan). Samples were prepared by depositing a droplet of the microsphere suspension onto a carbon-coated copper grid, followed by air-drying. Imaging was performed in bright-field mode, and selected area diffraction (SAD) was used to determine crystal structure.

#### 2.1.5. Fourier Transform Infrared Spectroscopy (FTIR)

Functional groups were characterized by Fourier transform infrared spectroscopy (FTIR, DA8.3, Bomem, Quebec, QC, Canada) in transmission mode, over a spectral range of 450–4000 cm^−1^. Analytical samples were prepared by mixing HAp powders with potassium bromide (KBr) at a ratio of 5:100.

#### 2.1.6. Inductively Coupled Plasma Mass Spectrometry (ICP-MS)

The elemental composition of Ca and P was quantified using inductively coupled plasma mass spectrometry (ICP-MS, Pe-Scies Elan 6100 DRC, Waltham, MA, USA). Additional trace element analysis followed ASTM F1185-03 guidelines. Briefly, 1 g of HAp powder was dissolved in 30 mL of 5% HCl, and the resulting solution was analyzed.

#### 2.1.7. Thermal Analysis

Thermal behavior of the composite microspheres and gelatin was evaluated using thermogravimetric analysis (TGA) and differential scanning calorimetry (DSC) (STA-409, Netzsch, Selb, Germany). Measurements were conducted from room temperature to 800 °C at a heating rate of 10 °C/min in air, monitoring weight loss and phase transformation.

#### 2.1.8. Heat Treatment

Based on the thermal analysis, synthesized G-HAM powders were subjected to annealing at 100 °C, 350 °C, 500 °C, 600 °C, and 700 °C for 1 h in air. Post-annealed samples were further examined using SEM, XRD, TEM, and FTIR to assess structural and compositional changes.

### 2.2. Drug Loading and Release Kinetics

#### 2.2.1. Antibiotic Loading on Composite Microspheres and Content Determination

In this study, several antibiotics commonly used for bone infections—vancomycin, teicoplanin, zyvox, and gentamicin—were selected for evaluation. G-HAM were immersed in antibiotic solutions of varying concentrations at 37 °C with gentle agitation (80 rpm) for 2 h, with a weight ratio of drug to G-HAM of 1/3. After loading, the microspheres were collected by centrifugation and air-dried at room temperature for 48 h.

Antibiotic quantification was performed using UV/VIS spectrophotometry (Hitachi U-3010, Tokyo, Japan) at the respective absorption maxima of each drug. Both vancomycin and teicoplanin were analyzed at 280 nm, with detection limits of 0.56 μg/mL and 0.50 μg/mL, respectively. Zyvox was measured at 252 nm, achieving a detection sensitivity of 0.07 μg/mL. Gentamicin determination required derivatization with o-phthalaldehyde reagent [42]. In this process, gentamicin, o-phthalaldehyde, and isopropanol were reacted at a 1:1:1 ratio for 45 min, and absorbance was subsequently recorded at 333 nm, with a detection threshold of 0.34 μg/mL.

#### 2.2.2. In Vitro Antibiotic Release from Microspheres

Drug release studies were carried out by dispersing 20 mg of antibiotic-loaded microspheres in 50 mL of phosphate-buffered saline (PBS, Sigma-Aldrich, St. Louis, MO, USA) contained in sealed vials. A phosphate-buffered saline (PBS) solution with a pH of 7.4 was used as the release medium in this study. PBS is frequently used to examine in vitro drug delivery behavior due to its stable, physiologically relevant ionic composition [43]. Moreover, the average physiological pH of the human body is approximately 7.4 [44]. The physicochemical behavior of the drug carrier is influenced by the composition of the dissolution medium, consequently impacting drug release kinetics and in vitro–in vivo correlations [45]. In addition, using PBS enables a more accurate observation of the intrinsic release kinetics and fitting behavior of the system, allowing clearer identification of the governing release model under controlled conditions. Samples were maintained in an orbital shaking water bath at 37 °C, rotating at 80 rpm. At predetermined time intervals, 1 mL of the release medium was withdrawn and replaced with an equal volume of fresh PBS to maintain sink conditions. The concentration of released antibiotics was analyzed following the same spectrophotometric protocols described above. All experiments were repeated in triplicate to ensure reproducibility.

### 2.3. Drug Release Kinetics

To investigate the release mechanism of antibiotics from G-HAM composite microspheres, three kinetic models commonly used in drug release studies were applied to fit the experimental data, including the first-order model, Higuchi model, and Korsmeyer–Peppas model [46,47].

#### 2.3.1. First-Order Kinetic Model

The first-order kinetic model assumes that the drug release rate is proportional to the remaining amount of drug in the system. It can be expressed as the following:

(2) $$\frac{dM}{dt}=-k_{1}M$$

where M is the remaining drug concentration, $k_{1}$ is the first-order rate constant, and $\frac{dM}{dt}$ represents the rate of drug depletion. This equation implies that the release rate decreases as the drug concentration decreases.

#### 2.3.2. Higuchi Model

The Higuchi model describes drug release from a homogeneous matrix as a diffusion process.

It assumes that the release rate is proportional to the square root of time and can be expressed as:

(3) $$M_{t}=k_{H}\sqrt{t}$$

where M is the cumulative amount of drug released at time *t* and k is the Higuchi dissolution constant.

This model is commonly used to describe diffusion-controlled release from solid or semi-solid matrices.

#### 2.3.3. Korsmeyer–Peppas Model

The Korsmeyer–Peppas model is an empirical equation used to analyze the mechanism of drug release from polymeric or composite matrices. It is expressed as the following:

(4) $$\frac{M_{t}}{M_{0}}=kt^{n}$$

where $M_{t}/M_{0}$ is the fraction of drug released at time t, k is the kinetic constant, and n indicates the underlying release mechanism, where n<0.43 corresponds to Fickian diffusion, 0.43<n<1 reflects anomalous (non-Fickian) transport, and n=1 represents zero-order release.

### 2.4. Antibacterial Assessment

To evaluate antibacterial activity, *Staphylococcus aureus*—a major pathogen responsible for severe bone infections—was selected as the test organism. The efficacy of the released antibiotics was examined using the standard agar diffusion assay [48]. *S. aureus* ATCC 6538P was first cultured in nutrient broth (10 g/L peptone, 2 g/L beef extract, and 5 g/L sodium chloride) at 37 °C under shaking conditions for 18 h, yielding a suspension of approximately 10^7^ CFU/mL. A 1.5 mL aliquot of this bacterial culture was then mixed into 250 mL of molten nutrient agar cooled to 45 °C before being poured into petri dishes.

For antibacterial testing, 70 μL of the elution solutions collected from the in vitro release studies were pipetted into wells at the center of agar plates, followed by incubation at 37 °C for 18 h. Antibacterial performance was assessed by measuring the diameter of the inhibition zones (mm) extending outward from the well edges. Negative controls, consisting of eluates from microspheres without antibiotic loading, were processed under identical conditions.

### 2.5. Cell Experiments

#### 2.5.1. Cell Culture

Human osteoblast-like G-292 cells obtained from the Bioresource Collection and Research Center (BCRC, Hsinchu, Taiwan; catalog no. BCRC 60225) were maintained in McCoy’s 5a medium (Sigma-Aldrich, St. Louis, MO, USA) supplemented with 10% fetal bovine serum (Biological Industries, Beit HaEmek, Israel), 100 U/mL penicillin, and 100 μg/mL streptomycin (Gibco, Waltham, MA, USA). Cells were incubated at 37 °C in a humidified atmosphere containing 5% CO_2_, with media refreshed every three days. For subculture, monolayers were rinsed with PBS, detached using 0.25% trypsin-EDTA (Gibco, Waltham, MA, USA), centrifuged, resuspended, and then used for subsequent experiments.

#### 2.5.2. Cytotoxicity Tests

The biocompatibility of G-HAM was assessed following ISO 10993-5 guidelines [49]. Extracts were prepared at a ratio of 0.2 g/mL in serum-containing culture medium and incubated for 24 h at 37 °C. Pure extracts and their dilutions (1:1 and 1:4) were applied to G-292 cells seeded at 1 × 10^4^ cells/well in 96-well plates. After 24 h of exposure, cell viability was determined by MTT assay. Medium alone served as the negative control, while phenol-containing medium acted as the positive control.

For direct-contact assays, sterilized G-HAM powders were placed into 24-well plates before seeding cells (1 × 10^4^ cells/well). Controls consisted of wells without powders. Cultures were maintained up to 14 days, with media replaced every three days. At designated intervals (days 1, 4, 7, and 14), cells were detached with trypsin-EDTA and evaluated using the trypan blue exclusion method. All experiments were performed in sextuplicate.

#### 2.5.3. MTT Assay

Cell metabolic activity was quantified via MTT assay. Briefly, after removing the culture medium and rinsing with PBS, 20 μL/well of MTT solution (0.5 mg/mL, Sigma M5655, St. Louis, MO, USA) was added and incubated for 4 h at 37 °C. The unreacted dye was replaced with 100 μL/well DMSO (Merck, Darmstadt, Germany) to dissolve formazan crystals under gentle shaking for 10 min. Absorbance was recorded at 540 nm using a microplate reader (Stat Fax-2100, Awareness Technologies, Palm City, FL, USA). Cell viability was expressed as a percentage relative to untreated controls.

#### 2.5.4. Cell Morphology

At selected time intervals, samples were fixed with 2.5% glutaraldehyde (Panreac, Castellar del Vallès, Spain) in 0.01 M PBS (pH 7.4) for 1 h, rinsed, and dehydrated through a graded ethanol series. After air-drying, specimens were sputter-coated with gold and observed using SEM.

### 2.6. Statistical Analysis

Drug-release and inhibition-zone experiments were performed in triplicate (*n* = 3), while the cell viability assay was conducted with six replicates (*n* = 6). Each replicate represents an independent measurement of the same sample under identical experimental conditions. The results obtained from repeated measurements were expressed as the mean values, and the error bars represent the standard deviation of the data. For the cell viability assay, statistical differences between each treatment group and the medium-only control were analyzed using the Student’s *t*-test in Microsoft Excel. A *p*-value of less than 0.05 was considered statistically significant.

## 3. Results and Discussion

### 3.1. Material Characterization

#### 3.1.1. Surface Observations

**Figure 1:**
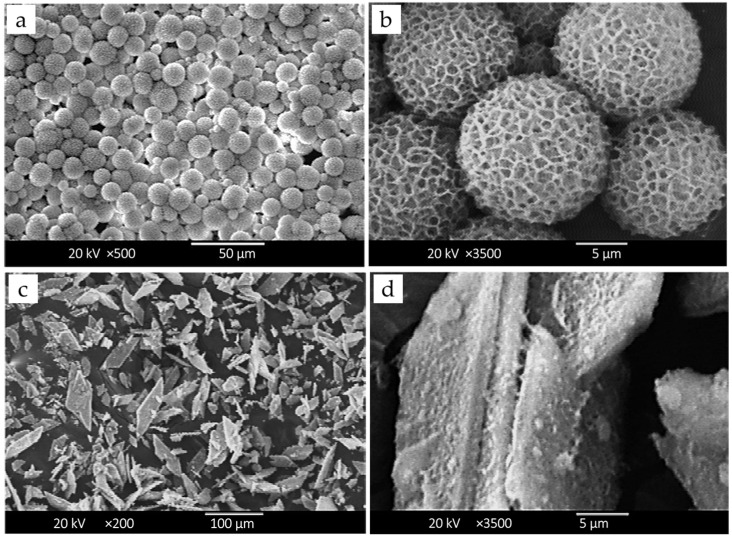
SEM micrographs showing the surface morphology of G-HAM at (**a**) low (×500) and (**b**) high (×3500) magnifications, and reference HAp at (**c**) low (×200) and (**d**) high (×3500) magnifications.

As shown in Figure 1a,b, the G-HAM exhibit a spherical morphology with a uniform size distribution of 5–10 μm. In contrast, reagent-grade HAp from Sigma-Aldrich (Figure 1c,d) displays irregular, polygonal, and flaky structures. Previous studies have reported that spherical particles are more favorable for osteo-conductivity and implantation [50].

**Figure 2:**
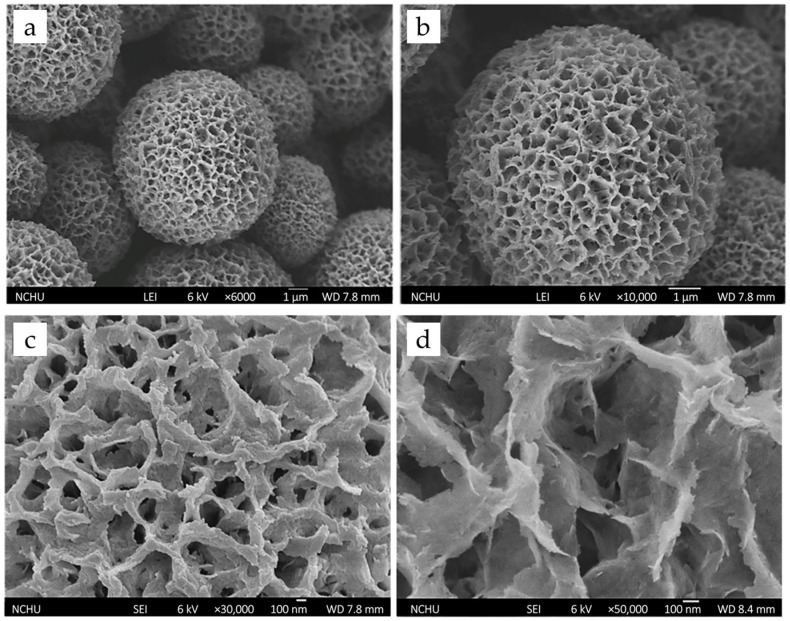
FESEM micrographs of G-HAM at different magnifications: (**a**) ×6000 and (**b**) ×10,000 showing overall spherical morphology, and (**c**) ×30,000 and (**d**) ×50,000 revealing detailed porous architecture.

The G-HAM also show rough, hydrangea-like surfaces with abundant pores, as clearly observed in the FE-SEM image (Figure 2). Surface topography is known to play a critical role in tissue response, drug release, and cellular activities. Hulbert et al. demonstrated that porous ceramics facilitated faster tissue healing and reduced fibrous encapsulation compared to non-porous structures, with interconnected pores further enabling vascularization [51]. Similarly, Chang et al. reported that porous HAp with pore structures was more osteoconductive and resorbable than dense HAp [52]. Moreover, Klose et al. showed that porosity enhances molecular mobility, while drug release rates decrease with increasing particle size [53]. Taken together, the homogeneous spherical geometry and highly porous architecture of the composite microspheres prepared in this study suggest excellent potential for antibiotic loading and accelerated bone ingrowth, making them highly promising for biomedical applications.

#### 3.1.2. XRD

**Figure 3:**
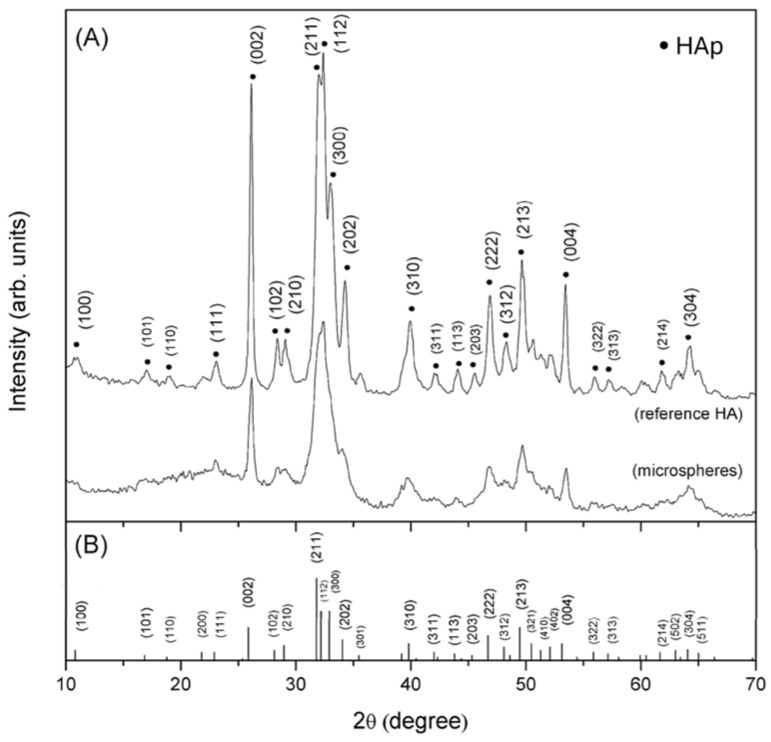
XRD patterns of (**A**) reference HAp and G-HAM, (**B**) JCPDS file No. 9-432.

The XRD patterns of reference HAp and G-HAM are shown in Figure 3. As illustrated in Figure 3A, both samples display similar peak positions, and the microspheres exhibit sharp peaks corresponding well with calcium phosphate hydroxide, Ca_5_(PO_4_)_3_(OH) [JCPDS 9-432], without additional phases (Figure 3B). This confirms that HAp powders are successfully synthesized via the wet-chemical method.

However, the microspheres display broader and weaker diffraction peaks, indicating lower crystallinity and/or smaller crystal size. During synthesis, dissociation of biopolymer chemical bonds allowed Ca^2+^ ions to interact with the anionic groups of the biopolymer, limiting the availability of free Ca^2+^ for HAp crystal growth. Consequently, the crystallization process was restricted. In addition, the merging of (211), (112), and (300) peaks in the microspheres suggests lattice distortion, a phenomenon also reported in previous studies due to interactions between the biopolymer backbone and the HAp lattice [54].

#### 3.1.3. FTIR Analysis

**Figure 4:**
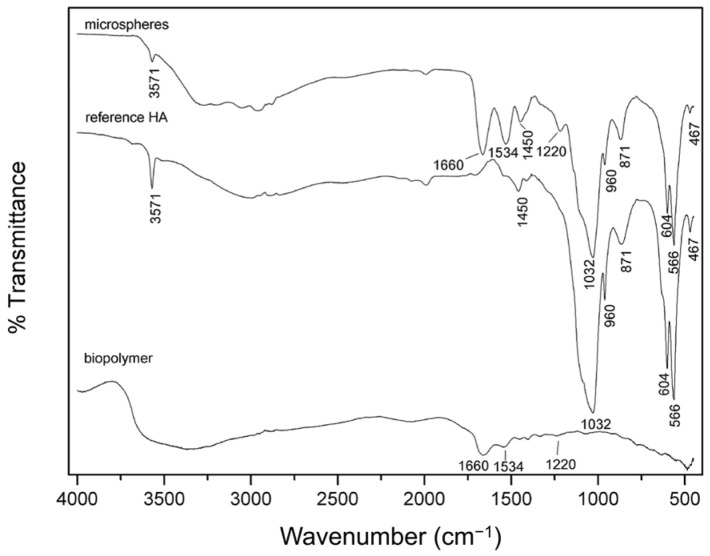
FTIR spectra of the biopolymer (gelatin), reference HAp, and G-HAM.

The FTIR spectra of reference HAp, G-HAM, and biopolymer are presented in Figure 4. For both reference HAp and G-HAM, characteristic phosphate (PO_4_^3−^) and hydroxyl (OH^−^) bands are clearly identified, including peaks at 467, 566, 604, 960, and 1032–1111 cm^−1^, consistent with reported HAp vibrational modes [55,56]. A broad band at 3100–3400 cm^−1^ corresponded to adsorbed hydrate, while a sharp peak at 3571 cm^−1^ indicated OH^−^ stretching.

Additional absorption bands at 871 and 1455 cm^−1^ suggest partial carbonate substitution, forming carbonated hydroxyapatite, which may also explain the slightly elevated Ca/P molar ratio observed by ICP-MS. The possibility of HPO_4_ contributions at 871 cm^−1^, as noted in previous studies, requires further thermal treatment analysis [57,58]. The pure biopolymer spectrum showed typical amide I, II, and III bands at 1660, 1534, and 1220 cm^−1^, along with additional peaks at 1442 and 1334 cm^−1^ attributed to amino acids [56,59]. In G-HAM, both HAp-related phosphate groups and biopolymer-associated amide bands (1660, 1550, 1220 cm^−1^) were detected, confirming successful incorporation of hydroxyapatite with the biopolymer.

The FTIR analysis was primarily conducted to confirm the compositional integrity of the synthesized G-HAM, specifically to verify the presence of both gelatin and hydroxyapatite (HAp) structures. The successful loading of antibiotics onto the microspheres, as well as their subsequent release behavior, was verified using UV–visible spectrophotometric measurements during the drug release study.

#### 3.1.4. Elemental Composition (ICP-MS)

The Ca and P contents of the synthesized G-HAM, measured by ICP-MS, are summarized in Table 1. The calculated Ca/P ratio is 1.69, which is close to the stoichiometric ratio of 1.67 corresponding to hydroxyapatite (Ca_10_(PO_4_)_6_(PO_4_)_6_(OH)_2_) in natural bone, ensuring proper phase formation and mimicking the mineral composition of biological apatite. In addition, the analysis of potential toxic elements (As, Cd, Hg, Pb) reveals concentrations far below the permissible limits specified by ASTM F1185–03—a widely recognized standard for hydroxyapatite ceramics used in surgical implants (not exceeding 3, 5, 5, and 10 ppm, respectively). Only trace amounts are detected, likely to originate from reagents or water. These results confirm that the prepared microspheres possess a bone-like Ca/P ratio and meet established safety standards for biomedical use.

#### 3.1.5. Thermal Analysis (TGA/DSC)

**Figure 5:**
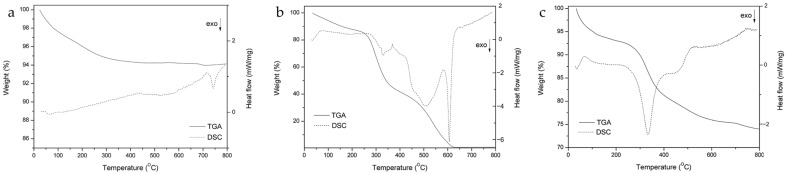
TGA and DSC curves of (**a**) reference HAp, (**b**) gelatin, and (**c**) G-HAM.

The TGA and DSC results of reference HAp, gelatin, and G-HAM are shown in Figure 5. Reference HAp exhibits minimal weight loss (6%) up to 800 °C, indicating high thermal stability, with only minor losses due to adsorbed water and volatiles.

As shown in Figure 5a, the continuous endothermic signal is observed across the heating range, and an exothermic peak appeared at 742 °C, suggesting crystal growth and/or structural adjustment. In contrast, pure gelatin (Figure 5b) underwent multiple decomposition stages, leading to complete weight loss finally. These changes correspond to water removal, amino acid degradation, and chain breakdown, with characteristic exothermic peaks in the DSC profile. Around 330 °C the weight loss of about 40% is ascribed to the condensation of OH and/or NH bonds. A sharp signal near 605 °C is attributed to the rapid combustion of residual carbon, marking the final degradation step.

For G-HAM (Figure 5c), the thermal behavior combined features of both HAp and gelatin. A more pronounced exothermic peak near 330 °C suggests more condensation between the polymer matrix and OH bonds of HAp, affecting heating stability. Overall weight loss is 26%, including 5% of initial evaporation of adsorbed H_2_O, which is greater than pure HAp but significantly lower than gelatin alone. Based on these data, the biopolymer content in the G-HAM was estimated at 21%, comparable to natural bone.

#### 3.1.6. Effect of Heat Treatment on Microspheres

**Figure 6:**
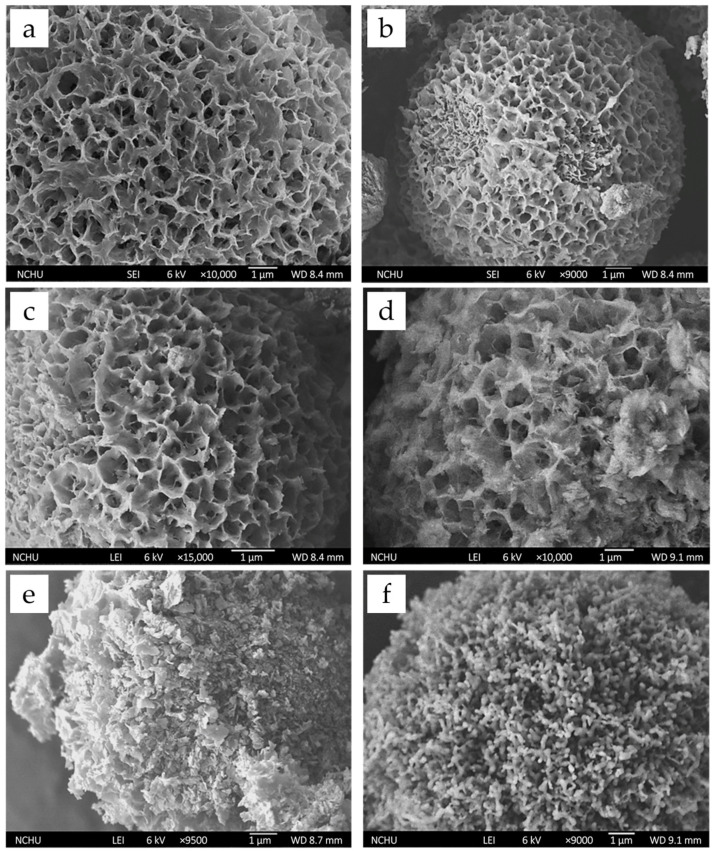
FE-SEM images of G-HAM subjected to various heat treatment conditions: (**a**) untreated, (**b**) 100 °C, (**c**) 350 °C, (**d**) 500 °C, (**e**) 600 °C, and (**f**) 700 °C.

To evaluate the influence of heating on crystallization and phase transformation, the microspheres were annealed at different temperatures based on the thermal analysis. As shown in Figure 6, the porous and spherical morphology remains stable below 500 °C. At 600 °C, partial surface collapse is observed, with pore structures beginning to close. When the temperature reached 700 °C, the porous flakes fully decomposed into nanoparticles, forming spheres with a coralline- and columnar-like surface and no visible pores. These changes suggest possible phase transformation at higher temperatures, which was further examined by XRD and FTIR analysis.

#### 3.1.7. Phase Transformation and Crystallinity After Heat Treatment

**Figure 7:**
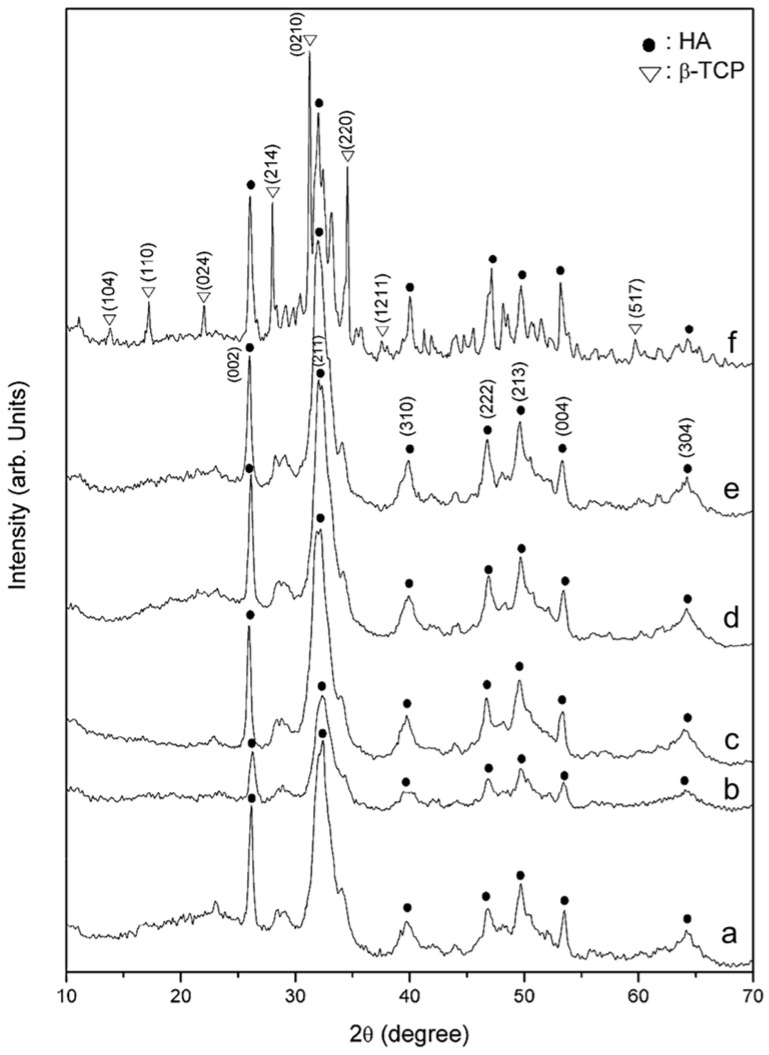
XRD analysis of G-HAM, showing phase evolution with increasing heat treatment temperature: (a) without heating, (b) 100 °C, (c) 350 °C, (d) 500 °C, (e) 600 °C, and (f) 700 °C.

The XRD spectra of G-HAM annealed at different temperatures are shown in Figure 7. Samples treated at and below 700 °C retain the characteristic diffraction peaks of HAp, located at 25.9, 31.8, 39.8, 46.8, 49.4, 53.3, and 64.2° (2θ) (JCPDS 9-432). At 700 °C, additional peaks corresponding to β-tricalcium phosphate (β-TCP, β-Ca_3_(PO_4_)_2_, JCPDS 09-0169) emerged, consistent with the morphological changes observed in Figure 6. The formation of this biphasic β-TCP/HAp structure is expected to alter the dissolution behavior of the microspheres, which may influence subsequent drug-release characteristics. Crystallite size, calculated using the Scherrer method, increases from 15.7 to 29.1 nm with higher annealing temperatures (Table 2), and the sharper diffraction peaks further confirm improved crystallinity.

**Figure 8:**
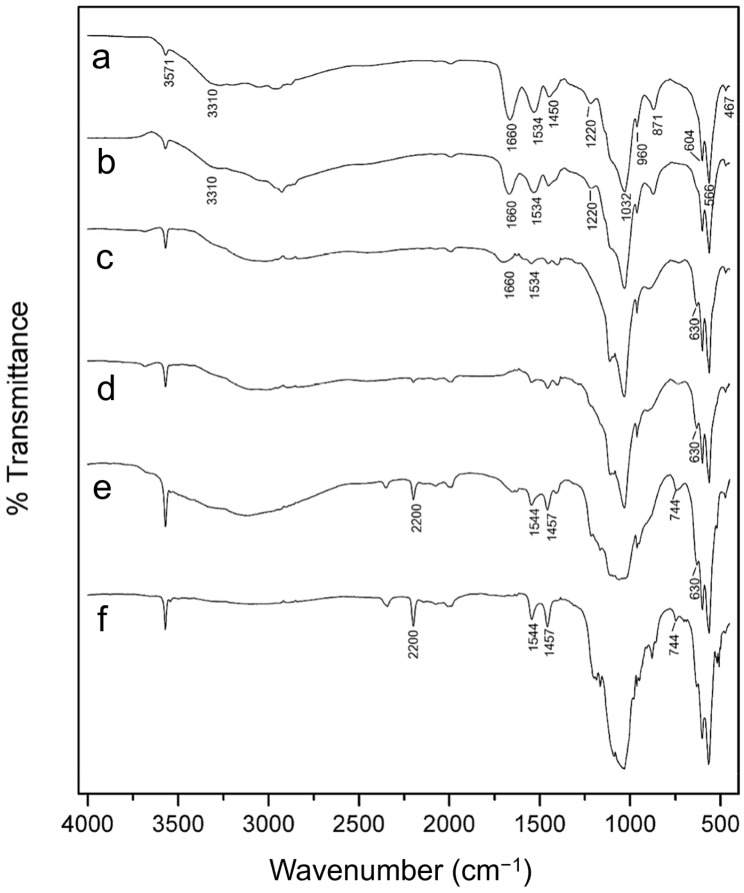
FTIR spectra of G-HAM, illustrating changes with increasing thermal treatment: (a) untreated, (b) 100 °C, (c) 350 °C, (d) 500 °C, (e) 600 °C, and (f) 700 °C.

The FTIR spectra of G-HAM annealed at various temperatures are shown in Figure 8. Characteristic biopolymer bands—including amide I (C=O stretching, 1660 cm^−1^), amide II (N–H deformation, 1534 cm^−1^), and amide III (N–H deformation, 1220 cm^−1^)—as well as the N–H stretching vibration at 3310 cm^−1^, are observed in samples without heat treatment and those treated at 100 and 350 °C. These functional groups gradually become less pronounced with increasing temperature, indicating progressive condensation reactions. At 500 °C, the disappearance of these peaks suggests the decomposition of gelatin, consistent with the major weight loss observed in its TGA/DSC curve (Figure 5b). Correspondingly, as shown in the SEM images (Figure 6), the hydrangea-like structure of G-HAM begins to collapse.

Meanwhile, phosphate-related HAp bands (500–1200 cm^−1^) become sharper with increasing temperature from 100 °C to 600 °C, and OH-related bands at 630 and 3571 cm^−1^ emerged, reflecting enhanced crystallization, corresponding to the improved crystallinity shown in Figure 6. A reduction in carbonate bands (871, 1450 cm^−1^) indicated decomposition, while a new band at 744 cm^−1^ confirms the formation of pyrophosphate species (P_2_O_7_^4−^), partly derived from HPO_4_^2−^ [58]. At higher temperatures such as 700 °C, OH bands weaken due to partial HAp-to-β-TCP transformation. Additional bands at 1457, 1544, and 2200 cm^−1^ are also detected at 600 and 700 °C, attributed to C=C and C≡N groups. This suggests that HAp plays a role in retarding the combustion of carbon-related fiber, compared with the total combustion of pure gelatin shown in Figure 5b.

#### 3.1.8. TEM Analysis of Composite Microspheres

**Figure 9:**
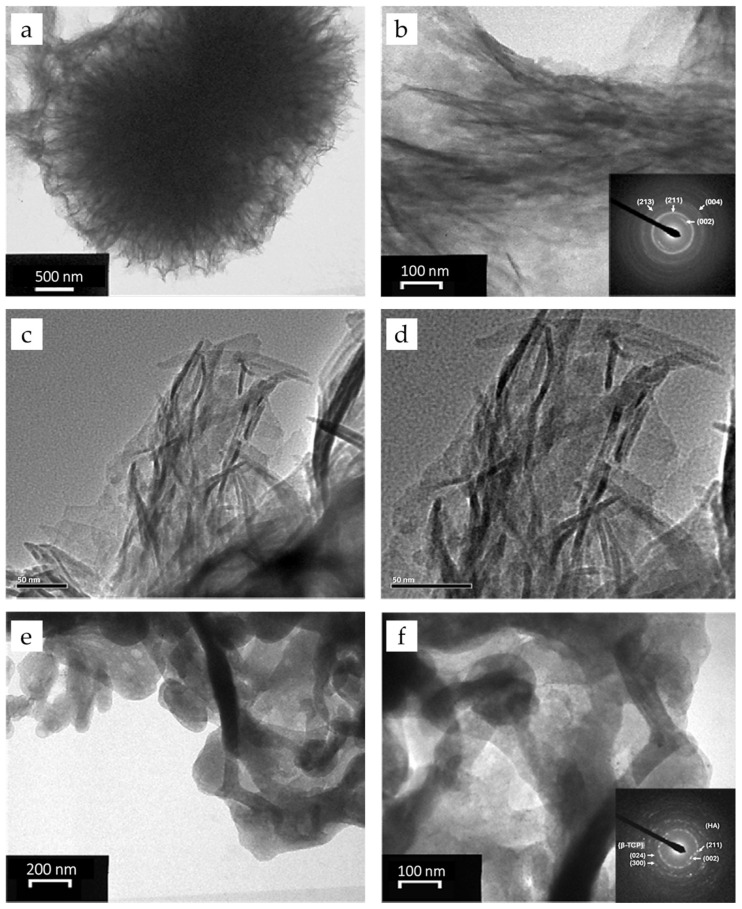
TEM analyses of G-HAM: Bright-field TEM micrographs at (**a**) low and (**b**) high magnifications, with the corresponding SAED pattern shown in the inset of (**b**); high-resolution TEM images recorded at magnifications of (**c**) 50,000× and (**d**) 80,000×; and bright-field TEM images of G-HAM after heat treatment at 700 °C, showing (**e**) low- and (**f**) high-magnification views, with the corresponding electron diffraction pattern presented in the inset of (**f**).

The crystalline structures of the composite microspheres were examined by TEM (Figure 9a–d). The nanocrystals exhibit a fibril-like morphology with diameters of 6 nm, and the corresponding selected area electron diffraction (SAED) patterns show diffraction rings indexed to the (211) and (213) planes of HAp, along with arc-shaped diffractions from the (002) and (004) planes. The strong arcs indicate a preferential orientation along the c-axis, consistent with XRD results and similar to the alignment of HAp with collagen fibers in natural bone [60]. TEM images also reveal that G-HAM are composed of aggregated fibrils, with HAp lattice structures present throughout, suggesting that the biopolymer did not disrupt HAp crystallinity.

After annealing at 700 °C (Figure 9e,f), the fibril edges transformed into particle-like structures. The arc-shaped diffraction features disappeared, and new diffraction rings corresponding to β-TCP ((024), (300)) appeared, confirming partial HAp-to-β-TCP transformation in agreement with XRD. The average fibril diameter increases to 25 nm, likely due to thermally driven aggregation and reduced surface energy.

### 3.2. Drug Release

**Figure 10:**
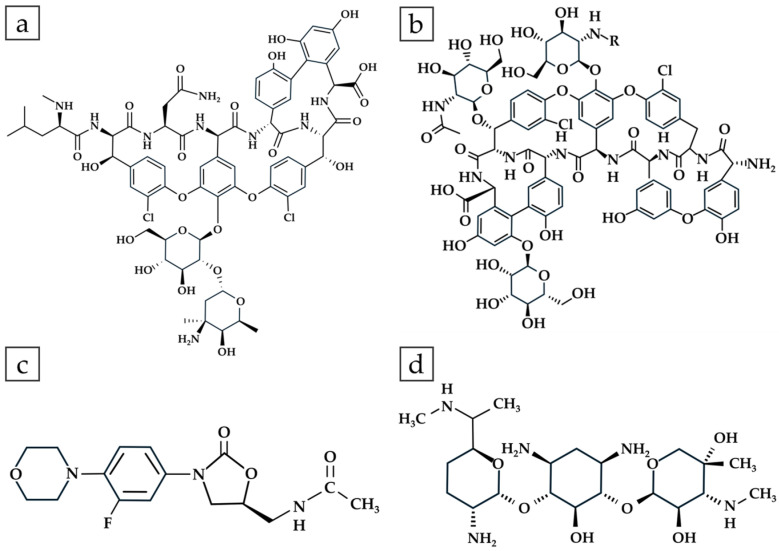
Chemical structures of (**a**) vancomycin, (**b**) teicoplanin, (**c**) zyvox, and (**d**) gentamicin.

**Figure 11:**
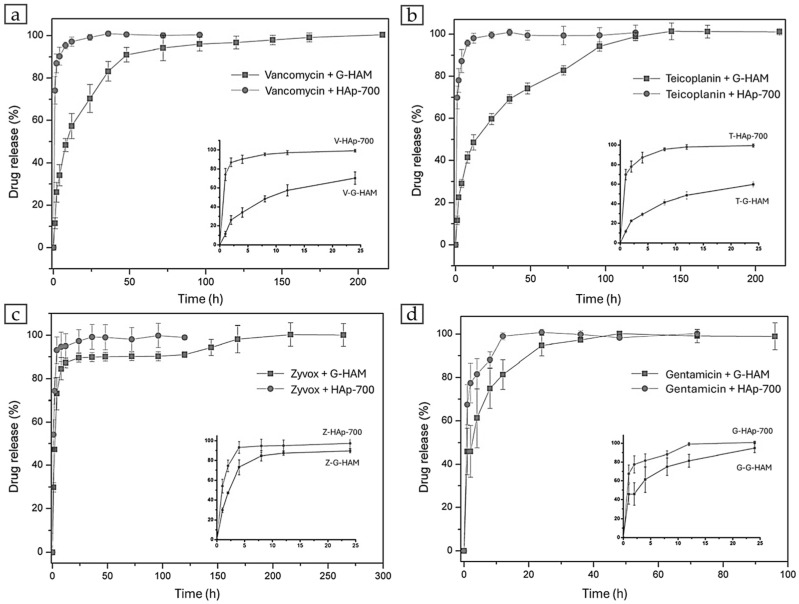
Drug release curves of (**a**) vancomycin, (**b**) teicoplanin, (**c**) zyvox, and (**d**) gentamicin loaded in untreated G-HAM and heat-treated microspheres at 700 °C (HAp-700), measured in phosphate-buffered saline at 37 °C. The inset in each graph shows the release profile within the first 24 h.

Four antibiotics—vancomycin, teicoplanin, zyvox, and gentamicin—were incorporated into G-HAM for in vitro release studies, with their structures shown in Figure 10. Four drug release profiles in PBS (pH 7.4) are presented in Figure 11 for both G-HAM untreated and G-HAM annealed at 700 °C (HAp-700). The drug release models are summarized in Table 3.

For G-HAM, all drugs exhibit an initial burst release followed by a more sustained release phase. The initial burst release is attributed to antibiotics adsorbed on the macropore surfaces, whereas the subsequent slower release phase likely results from gradual desorption and diffusion from the inner pores. Within the first 24 h, the cumulative release reaches approximately 70% for vancomycin, 60% for teicoplanin, 90% for zyvox, and 95% for gentamicin.

According to the kinetic fitting results, vancomycin and teicoplanin showed release behaviors consistent with the first-order model, suggesting that the release rate was primarily governed by interfacial reactions resulting from the water dissociation. These two antibiotics possess hydroxyl (–OH) groups and can form hydrogen bonds between drug molecules and with the G-HAM matrix, while the greater molecular weight can form the van der Waals force interactions. Consequently, stronger intermolecular forces contributed to their slower and more prolonged release profiles. In contrast, zyvox showed poor correlation with all three models, indicating a different release mechanism. Because zyvox lacks hydroxyl groups with the least molecular weight, resulting in the weaker intermolecular interactions, and revealing the greatest initial in 8 h. For gentamicin, the initial burst release followed the first-order kinetic model, indicating that it was governed by interfacial reaction control. However, the drug release in the first 24 h is the greatest one, since the intermolecular interactions are relatively weak due to fewer hydroxyl groups and a lower molecular weight than vancomycin and teicoplanin.

**Figure 12:**
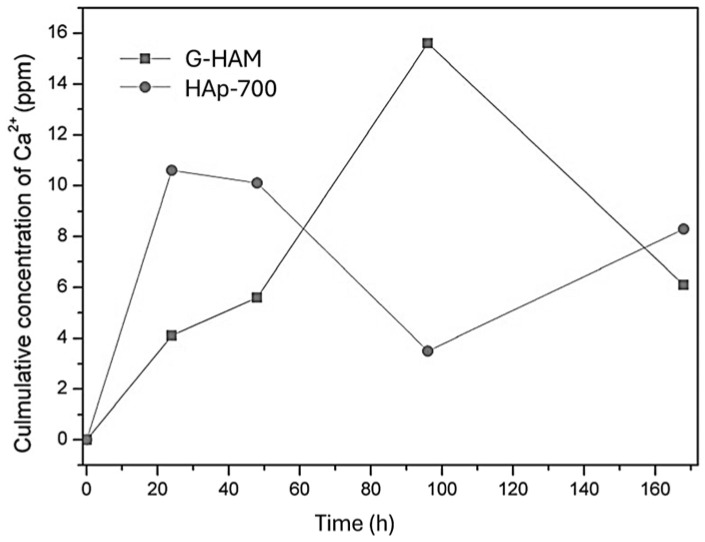
Cumulative Ca^2+^ ion concentrations released from untreated G-HAM without heat treatment and those calcined at 700 °C (HAp-700) during immersion in PBS (pH 7.4).

Figure 11 also demonstrates that the HAp-700 microspheres display markedly faster release kinetics, achieving over 95% cumulative release within 20 h (complete release of vancomycin, teicoplanin, and zyvox within 24 h, and gentamicin within 12 h). This difference can be attributed to both phase and morphological changes induced by annealing. Figure 5c indicates that HAp-700 microspheres lost their porous structure and contained significant amounts of β-TCP, whose dissolution is known to accelerate drug release [61]. To further verify the effect of phase composition on the release behavior, dissolution experiments using drug-unloaded G-HAM were conducted under the same conditions as the release tests, as shown in Figure 12.

The results confirmed the burst increasing Ca^2+^ concentration in PBS for the initial 48 h, reflecting the higher solubility of HAp-700, approximately twice that of G-HAM. In addition, the decrease in hydroxyl groups (–OH) during annealing reduced the potential for chemical bonding between the antibiotic molecules and β-TCP, also contributing to the faster release observed in HAp-700.

Overall, the composite microspheres demonstrate an initial burst—useful for immediate sterilization—followed by a prolonged release phase that could prevent infection. These results suggest strong potential for application in bone tissue engineering. The formation of the biphasic β-TCP/HAp composite facilitates faster drug diffusion. This indicates that heat treatment provides an effective approach to tune the crystalline phase composition of G-HAM and, consequently, to modulate their drug-release kinetics.

It should be noted that the drug release tests in this study were performed in PBS to maintain controlled conditions for kinetic analysis. While PBS provides a standardized environment, it does not fully represent physiological complexity. Future work will include release studies in more biologically relevant media, such as simulated body fluid (SBF), Hank’s balanced salt solution (HBSS), or protein-containing media (e.g., serum or albumin solutions), to better evaluate antibiotic release from G-HAM under physiologically representative environments.

### 3.3. Inhibition Zone Observations

**Figure 13:**
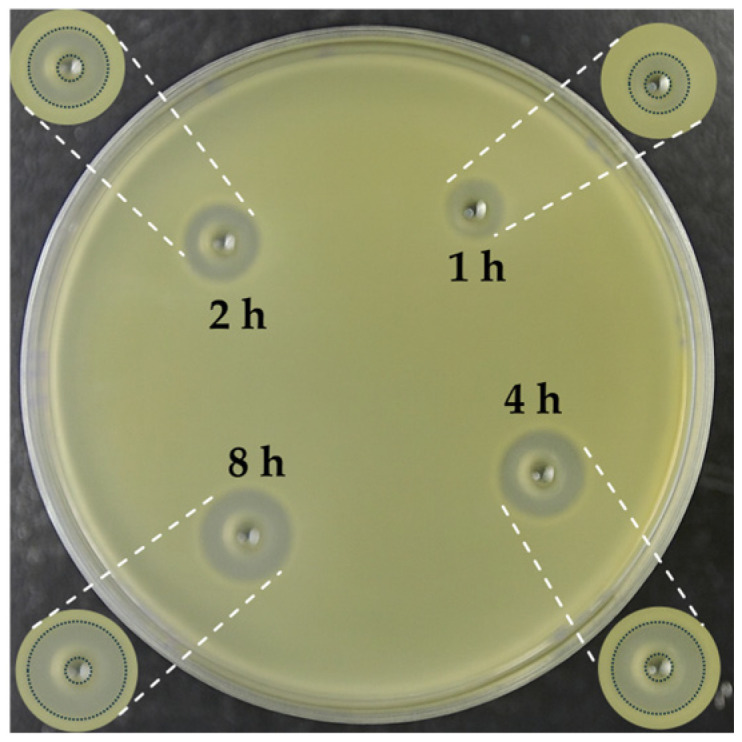
Antibacterial activity illustrated by clear inhibition zones around wells loaded with 70 μL of teicoplanin-containing elution solutions obtained from G-HAM at varying release times (1, 2, 4, and 8 h) and the inhibition zones outlined with dotted lines for easier visualization.

For reference, Figure 13 illustrates a bacterial inhibition zone produced by teicoplanin released from microspheres at different exposure times. The clear circular zones indicate regions without bacterial growth. No inhibition is observed for control samples (microspheres without antibiotic loading), confirming that antibacterial activity is solely due to drug release.

**Figure 14:**
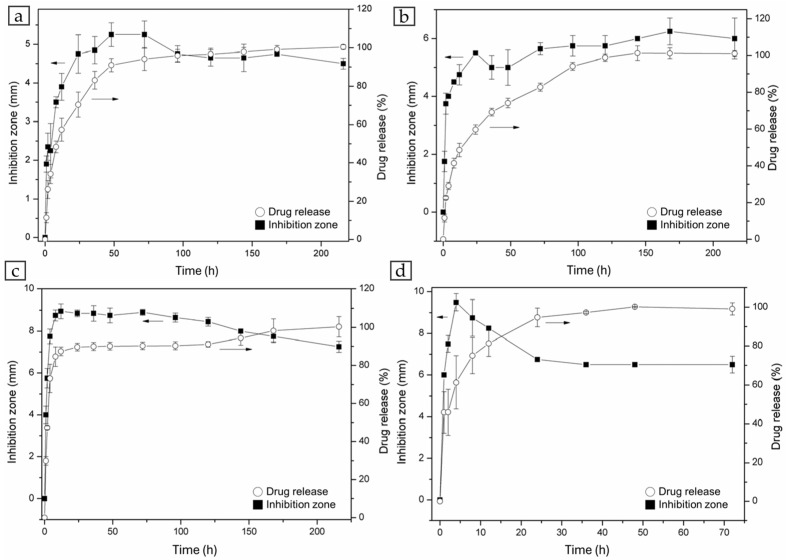
Correlation of inhibition zone diameter with cumulative (**a**) vancomycin, (**b**) teicoplanin, (**c**) zyvox, and (**d**) gentamicin concentration released from G-HAM into PBS solution.

The inhibition zones against *Staphylococcus aureus* for all tested antibiotics are presented in Figure 14, consistent with the release profiles in Figure 11. Vancomycin exhibits a gradual increase in inhibition zone diameter, continuing to expand up to approximately 75 h before declining. Teicoplanin shows the most persistent antibacterial effect, with inhibition zones steadily enlarging throughout the observation period. Zyvox maintains a relatively stable inhibition zone and then gradually decreases, while gentamicin demonstrates the sharpest decline in antibacterial activity, as its release is nearly complete within two days, leading to the rapid decrease in the inhibiting zone.

Overall, zyvox and gentamicin, which exhibit faster release (about 90% and 95% within 24 h), show strong antibacterial activity at the early stage; however, their inhibition zones gradually diminish and eventually fall below the corresponding release curves, indicating that although drug release continues, the concentration becomes insufficient to maintain prolonged inhibition. This behavior is attributed to the high initial burst release, which generates large inhibition zones (approximately 8–9 mm within the first 24 h) but rapidly depletes the available drug, resulting in reduced long-term efficacy. In contrast, teicoplanin and vancomycin, releasing about 60% and 70% of their load within the first 24 h, respectively, form smaller initial inhibition zones (about 4–5 mm), while the former maintains bacterial suppression for a much more extended period.

The inhibition zones correlated well with the measured drug concentrations. The minimal inhibitory concentrations (MICs) of vancomycin, zyvox, teicoplanin, and gentamicin against *S. aureus* were 1, 2, 2, and 8 mg/L, respectively [62]. At their MICs, vancomycin produces no detectable inhibition zone, whereas zyvox, teicoplanin, and gentamicin generate zones of 2.5, 1.5, and 1.5 mm, respectively. The inhibition zones observed after 1 h of release from the microspheres exceed these MIC-based values, indicating that the antibiotic concentrations achieved within 50 mL of medium are sufficient to suppress bacterial growth within 1 h.

The agar diffusion tests confirm that all antibiotics retain their activity after release and achieve concentrations exceeding their MIC values. In addition, the antibiotic loading was performed at 37 °C because the intended application of the composite microspheres is in the human body, where 37 °C represents the physiological temperature. These findings indicate that the antibiotics encapsulated within G-HAM remain chemically stable, biologically active, and effective for potential applications in bone infection treatment.

### 3.4. Cell Experiment

**Figure 15:**
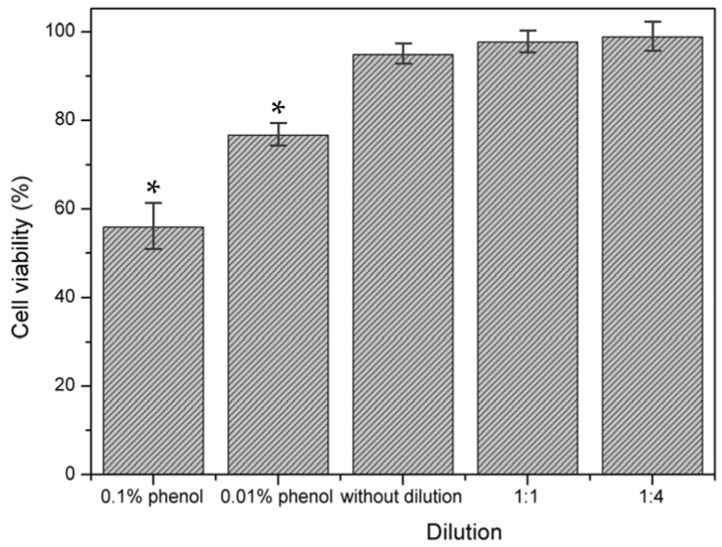
In vitro cytotoxicity assay performed according to ISO 10993-5 guidelines. Cells were treated with material extracts either undiluted or diluted (1:1 and 1:4) for 24 h. Culture medium alone served as the negative control (100% viability), while phenol-containing medium (0.1% and 0.01%) was used as the positive control (*n* = 6). * Statistically significant difference between the negative control and other groups.

Cytotoxicity of G-HAM was evaluated according to ISO 10993-5. Powders were incubated in culture medium for 24 h, and the resulting extracts (undiluted, 1:1, and 1:4 dilutions) were applied to osteoblast-like cells, with complete medium as the negative control and phenol-containing medium as the positive control. Quantitative results from the MTT assay (Figure 15) show that the positive control (0.1% phenol) reduced cell viability by 46%, while 0.01% phenol caused a 23% reduction. In contrast, microsphere extracts exhibit only minor effects, with 5%, 2%, and 1% reduction in cell viability for the undiluted, 1:1, and 1:4 dilutions, respectively, confirming their non-toxic behavior and suitability for tissue engineering.

**Figure 16:**
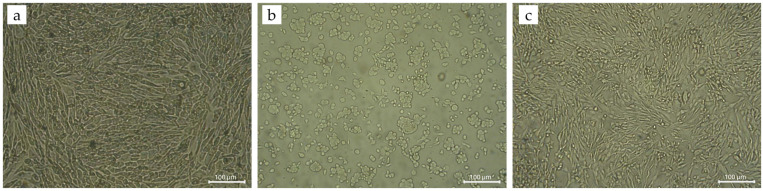
Representative microscopic images of cells after 24 h incubation at 100× magnification. (**a**) Negative control: complete culture medium. (**b**) Positive control: medium supplemented with 0.1% phenol. (**c**) Undiluted material extract.

Morphological observations under optical microscopy further confirm these results. As shown in Figure 16a, the negative control exhibits a confluent monolayer of evenly distributed osteoblast-like cells with normal morphology. In contrast, the positive control (Figure 16b) shows disrupted growth, irregular cell shapes, open areas between cells, and abnormal cytoplasmic features, indicating extensive cell lysis. Cells exposed to undiluted extracts (Figure 16c) maintain normal morphology and confluence, closely resembling the negative control (Figure 16a), and no visible damage is observed at diluted extract concentrations. Both quantitative and qualitative assessments demonstrate that G-HAM do not induce cytotoxic effects, with osteoblast-like cells maintaining high viability and normal morphology. These findings, summarized in Table 4, are consistent with ISO 10993-5 guidelines [49,63], further supporting the biocompatibility of the material for potential biomedical applications.

**Figure 17:**
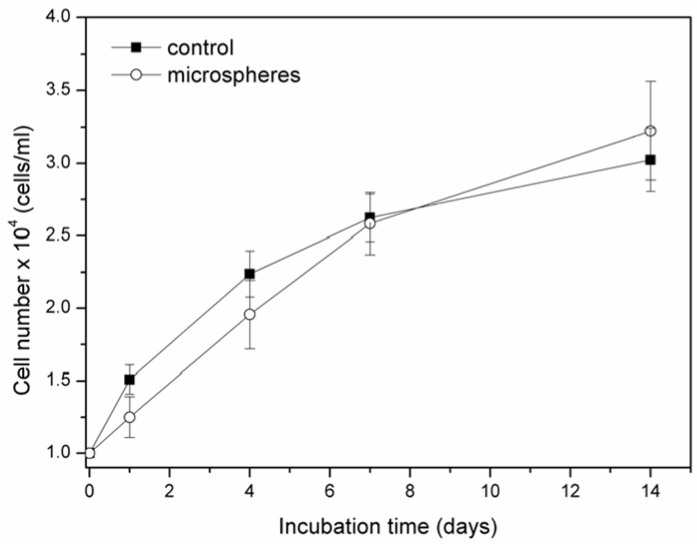
Proliferation of osteoblast-like cells over time when cultured in the presence of G-HAM compared with the control group.

In the direct contact method, osteoblast-like cells were cultured in the presence of G-HAM, and viability was assessed using the trypan blue exclusion assay. As shown in Figure 17, cell proliferation increased over time and, although slightly lower than the control (tissue culture plate) during the early stages, surpassed the control by day 14, increasing from 10^4^ to 3 × 10^4^ cells/cm^2^. This indicates that G-HAM supprt long-term cell growth but do not adversely affect viability.

**Figure 18:**
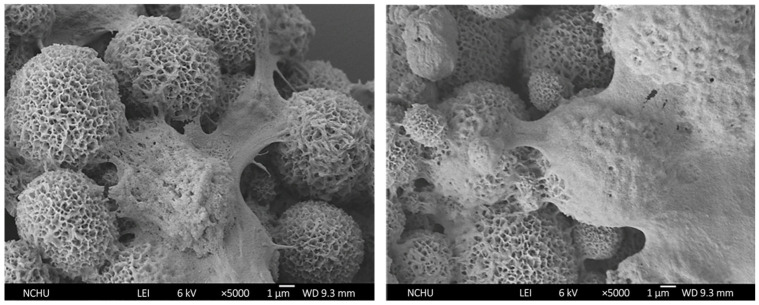
FESEM micrographs observed at two areas at ×5000, showing osteoblast-like cells adhered to the microsphere surfaces after 14 days of culture.

FESEM images (Figure 18) further confirmed favorable interactions between cells and G-HAM. Osteoblast-like cells adhered well, forming confluent monolayers with polygonal or elongated morphology. Filopodia extended across surfaces and into pores, bridging adjacent microspheres and demonstrating active attachment. These observations suggest that G-HAM exhibit excellent biocompatibility and bioactivity.

## 4. Conclusions

Composite microspheres consisting of the bioactive ceramic hydroxyapatite (Ca_10_(PO_4_)_6_(OH)_2_, HAp) and a biodegradable polymer gelatin were successfully fabricated using a wet-chemical method. SEM analysis confirmed that the gelatin-HAp composite microspheres (G-HAM) exhibited a porous architecture. ICP-MS results indicated a Ca/P ratio of 1.69, closely matching the 1.67 value typical of human bone, while concentrations of trace elements were well below the ASTM 1185-03 limits. XRD and FTIR analyses verified the coexistence of inorganic and organic phases; however, partial conversion of HAp to β-TCP occurred upon annealing at 700 °C. In vitro release studies demonstrated that vancomycin- and teicoplanin-loaded G-HAM achieved more prolonged release than zyvox and gentamicin, attributed to their greater molecular weight and strong binding of calcium ions by carboxyl groups in their molecular structures. Compared with annealed G-HAM, the as-prepared porous G-HAM showed slower drug release, highlighting the role of porosity in sustaining antibiotic delivery. Moreover, heat treatment at 700 °C resulted in the formation of a β-TCP/HAp composite that significantly enhanced the drug-release rate, demonstrating the potential of thermal processing as a practical means for tuning antibiotic delivery profiles. The relationship between release kinetics and antibacterial behavior is also evident. Antibiotics with a higher initial burst, such as zyvox and gentamicin, produce larger inhibition zones initially but then gradually decrease. In contrast, vancomycin and teicoplanin, which exhibit a more gradual release, maintain smaller but longer-lasting inhibition zones. These findings confirm that the encapsulated antibiotics remain chemically stable and biologically active after release, achieving concentrations exceeding the minimum inhibitory concentration within 1 h in 50 mL PBS. Cytotoxicity evaluation further showed that G-HAM were biocompatible and bioactive. Osteoblast-like cells adhered well, spread effectively, and formed confluent monolayers on their surfaces. These results suggest that the composite microspheres are promising candidates for drug carriers and hard tissue engineering.

## Figures and Tables

**Table 1:** Elemental analysis for G-HAM by ICP-MS.

Element	Concentration (ppm)
P	4640
Ca	7869
As	0.04
Cd	0.06
Hg	0.01
Pb	0.23

**Table 2:** Grain size of G-HAM at different heat treatments.

Heat Treatment Temperature (°C)	Grain Size (nm)
Microspheres without heat treatment	19.41
100 °C	15.68
350 °C	20.39
500 °C	25.47
600 °C	26.30
700 °C	29.11

**Table 3:** Correlation coefficients (R^2^) of different kinetic models for the release of antibiotics from G-HAM at various time intervals.

		R^2^		
Antibiotic	Time	1st Order	Higuchi’s	Korsmeyer-Peppas’s
Vancomycin	In 24 h	0.9457	0.9605	0.9448
	After 24 h	0.9732	0.7859	0.857
Teicoplanin	In 24 h	0.9203	0.9574	0.946
	After 24 h	0.9562	0.8131	0.8954
Zyvox	In 24 h	0.7315	0.7526	0.863
	After 24 h	0.7328	0.9091	0.8359
Gentamicin	In 24 h	0.9973	0.9644	0.9635
	After 24 h	0.8887	0.9192	0.9355

**Table 4:** The summary of the cytotoxicity test.

Cellular Response	Negative (Medium Without Material Extracts)	Positive Control (Medium Containing 0.1% Phenol Solution)	Material Extract (Undiluted Extract)
confluency	Normal	Cannot be observed	Normal
Cell membrane lysis	Not existing	Existing	Not existing
Aggregation	Not existing	Existing	Not existing
Granulation	Not existing	Existing	Not existing
Paraquat toxicity	Not existing	Existing	Not existing

## Data Availability

Data are contained within the article.

## References

[1] Cuylear D.L., Elghazali N.A., Kapila S.D., Desai T.A. (2023). Calcium Phosphate Delivery Systems for Regeneration and Biomineralization of Mineralized Tissues of the Craniofacial Complex. Mol. Pharm..

[2] Zhang Y., Shu T., Wang S., Liu Z., Cheng Y., Li A., Pei D. (2022). The Osteoinductivity of Calcium Phosphate-Based Biomaterials: A Tight Interaction With Bone Healing. Front. Bioeng. Biotechnol..

[3] Montesissa M., Tommasini V., Rubini K., Boi M., Baldini N., Boanini E. (2025). State of Art and Perspective of Calcium Phosphate-Based Coatings Coupled with Bioactive Compounds for Orthopedic Applications. Nanomaterials.

[4] Wu M.-Y., Kao I.F., Fu C.-Y., Yen S.-K. (2023). Effects of Adding Chitosan on Drug Entrapment Efficiency and Release Duration for Paclitaxel-Loaded Hydroxyapatite—Gelatin Composite Microspheres. Pharmaceutics.

[5] Wu M.-Y., Liang Y.-H., Yen S.-K. (2022). Effects of Chitosan on Loading and Releasing for Doxorubicin Loaded Porous Hydroxyapatite–Gelatin Composite Microspheres. Polymers.

[6] Ebrahimi S., Stephen Sipaut@ Mohd Nasri C., Bin Arshad S.E. (2021). Hydrothermal synthesis of hydroxyapatite powders using Response Surface Methodology (RSM). PLoS ONE.

[7] Matamoros-Veloza Z., Rendon-Angeles J.C., Yanagisawa K., Ueda T., Zhu K., Moreno-Perez B. (2021). Preparation of Silicon Hydroxyapatite Nanopowders under Microwave-Assisted Hydrothermal Method. Nanomaterials.

[8] Castro M.A.M., Portela T.O., Correa G.S., Oliveira M.M., Rangel J.H.G., Rodrigues S.F., Mercury J.M.R. (2022). Synthesis of hydroxyapatite by hydrothermal and microwave irradiation methods from biogenic calcium source varying pH and synthesis time. Boletín Soc. Española Cerámica Vidr..

[9] Mohd Pu’ad N.A.S., Alipal J., Abdullah H.Z., Idris M.I., Lee T.C. (2021). Synthesis of eggshell derived hydroxyapatite via chemical precipitation and calcination method. Mater. Today Proc..

[10] Jang J.-H., Oh B., Lee E.-J. (2021). Crystalline hydroxyapatite/graphene oxide complex by low-temperature sol-gel synthesis and its characterization. Ceram. Int..

[11] Jaafar A., Schimpf C., Mandel M., Hecker C., Rafaja D., Krüger L., Arki P., Joseph Y. (2022). Sol–gel derived hydroxyapatite coating on titanium implants: Optimization of sol–gel process and engineering the interface. J. Mater. Res..

[12] Catauro M., Barrino F., Blanco I., Piccolella S., Pacifico S. (2020). Use of the Sol–Gel Method for the Preparation of Coatings of Titanium Substrates with Hydroxyapatite for Biomedical Application. Coatings.

[13] Mousavi M.S., Pourmadadi M., Abdouss M., Rahdar A., Fathi-Karkan S., Pandey S. (2024). Gelatin/CMC/HAP Nanocomposites Based on Double Micro-emulsion for Delivery of 5-FU: Synthesis and Chemical–Physical Characterization. BioNanoScience.

[14] Sadat Nasiri S., Pourmadadi M., Rahdar A., Pandey S. (2024). Gelatin/PVP/hydroxyapatite nanocomposite based on double micro-emulsion for tissue engineering applications. J. Mol. Liq..

[15] Fan J., Abedi-Dorcheh K., Sadat Vaziri A., Kazemi-Aghdam F., Rafieyan S., Sohrabinejad M., Ghorbani M., Rastegar Adib F., Ghasemi Z., Klavins K. (2022). A Review of Recent Advances in Natural Polymer-Based Scaffolds for Musculoskeletal Tissue Engineering. Polymers.

[16] Wu M.-Y., Huang S.-W., Kao I.F., Yen S.-K. (2024). The Preparation and Characterization of Chitosan/Calcium Phosphate Composite Microspheres for Biomedical Applications. Polymers.

[17] Liu W., Cheong N., He Z., Zhang T. (2025). Application of Hydroxyapatite Composites in Bone Tissue Engineering: A Review. J. Funct. Biomater..

[18] Yu F., Wang Q., Liu D., Fan X., Tong L., Shen G., Zhai F. (2024). Studies of a novel nano sustained-released drug delivery system with a hydroxyapatite core and polysuccinimide coating structure. Mater. Adv..

[19] Xing F., Chi Z., Yang R., Xu D., Cui J., Huang Y., Zhou C., Liu C. (2021). Chitin-hydroxyapatite-collagen composite scaffolds for bone regeneration. Int. J. Biol. Macromol..

[20] Kavitha Sri A., Arthi C., Neya N.R., Hikku G.S. (2023). Nano-hydroxyapatite/collagen composite as scaffold material for bone regeneration. Biomed. Mater..

[21] Ciobanu C.S., Iconaru S.L., Predoi D., Trușcă R.-D., Prodan A.M., Groza A., Chifiriuc M.C., Beuran M. (2021). Fabrication of Novel Chitosan–Hydroxyapatite Nanostructured Thin Films for Biomedical Applications. Coatings.

[22] Benedini L., Laiuppa J., Santillán G., Baldini M., Messina P. (2020). Antibacterial alginate/nano-hydroxyapatite composites for bone tissue engineering: Assessment of their bioactivity, biocompatibility, and antibacterial activity. Mater. Sci. Eng. C.

[23] Sadeghianmaryan A., Naghieh S., Yazdanpanah Z., Alizadeh Sardroud H., Sharma N.K., Wilson L.D., Chen X. (2022). Fabrication of chitosan/alginate/hydroxyapatite hybrid scaffolds using 3D printing and impregnating techniques for potential cartilage regeneration. Int. J. Biol. Macromol..

[24] Mahmoud E.M., Sayed M., El-Kady A.M., Elsayed H., Naga S.M. (2020). In vitro and in vivo study of naturally derived alginate/hydroxyapatite bio composite scaffolds. Int. J. Biol. Macromol..

[25] Wu M.-Y., Kao I.F., Yen S.-K. (2025). Effects of Chitosan on Drug Load and Release for Cisplatin–Hydroxyapatite–Gelatin Composite Microspheres. Polymers.

[26] Sharifi S., Zaheri Khosroshahi A., Maleki Dizaj S., Rezaei Y. (2022). Preparation, Physicochemical Assessment and the Antimicrobial Action of Hydroxyapatite–Gelatin/Curcumin Nanofibrous Composites as a Dental Biomaterial. Biomimetics.

[27] Mobika J., Rajkumar M., Nithya Priya V., Linto Sibi S.P. (2021). Effect of chitosan reinforcement on properties of hydroxyapatite/silk fibroin composite for biomedical application. Phys. E Low-Dimens. Syst. Nanostruct..

[28] Mobika J., Rajkumar M., Nithya Priya V., Linto Sibi S.P. (2020). Substantial effect of silk fibroin reinforcement on properties of hydroxyapatite/silk fibroin nanocomposite for bone tissue engineering application. J. Mol. Struct..

[29] Nichol T., Callaghan J., Townsend R., Stockley I., Hatton P.V., Le Maitre C., Smith T.J., Akid R. (2021). The antimicrobial activity and biocompatibility of a controlled gentamicin-releasing single-layer sol-gel coating on hydroxyapatite-coated titanium. Bone Jt. J..

[30] Tun T., Hnin H.M., Kanchanasin P., Phongsopitanun W., Nalinratana N., Jansook P. (2025). Development of Eudragit^®^-coated linezolid-loaded lipid nanoparticles for enhanced ocular delivery in bacterial keratitis treatment. Colloids Surf. A Physicochem. Eng. Asp..

[31] Amiri N., Ajami S., Shahroodi A., Jannatabadi N., Amiri Darban S., Fazly Bazzaz B.S., Pishavar E., Kalalinia F., Movaffagh J. (2020). Teicoplanin-loaded chitosan-PEO nanofibers for local antibiotic delivery and wound healing. Int. J. Biol. Macromol..

[32] Gao X., Xu Z., Li S., Cheng L., Xu D., Li L., Chen L., Xu Y., Liu Z., Liu Y. (2023). Chitosan-vancomycin hydrogel incorporated bone repair scaffold based on staggered orthogonal structure: A viable dually controlled drug delivery system. RSC Adv..

[33] Alegrete N., Sousa S.R., Padrão T., Carvalho Â., Lucas R., Canadas R.F., Lavrador C., Alexandre N., Gärtner F., Monteiro F.J. (2023). Vancomycin-Loaded, Nanohydroxyapatite-Based Scaffold for Osteomyelitis Treatment: In Vivo Rabbit Toxicological Tests and In Vivo Efficacy Tests in a Sheep Model. Bioengineering.

[34] Huang C.-L., Fang W., Huang B.-R., Wang Y.-H., Dong G.-C., Lee T.-M. (2020). Bioactive Glass as a Nanoporous Drug Delivery System for Teicoplanin. Appl. Sci..

[35] Chaves B.J., Tadi P. (2025). Gentamicin.

[36] Thapa R., Pandey P., Parat M.-O., Gurung S., Parekh H.S. (2025). Intravaginal application of linezolid-infused sol-gel for prophylaxis and treatment of sexually transmitted infections. J. Pharm. Investig..

[37] Klicova M., Mullerova S., Rosendorf J., Klapstova A., Jirkovec R., Erben J., Petrzilkova M., Raabová H., Šatínský D., Melicherikova J. (2023). Large-Scale Development of Antibacterial Scaffolds: Gentamicin Sulfate-Loaded Biodegradable Nanofibers for Gastrointestinal Applications. ACS Omega.

[38] Motasadizadeh H., Tavakoli M., Damoogh S., Mottaghitalab F., Gholami M., Atyabi F., Farokhi M., Dinarvand R. (2022). Dual drug delivery system of teicoplanin and phenamil based on pH-sensitive silk fibroin/sodium alginate hydrogel scaffold for treating chronic bone infection. Biomater. Adv..

[39] Zhang P., Sun Y., Yang H., Liu D., Zhang F., Zhang Y., Zhong W., Zuo B., Zhou Z. (2023). Vancomycin-loaded silk fibroin microspheres in an injectable hydrogel for chronic osteomyelitis therapy. Front. Bioeng. Biotechnol..

[40] Kai K.C., Borges R., Pedroni A.C.F., Pelosine A.M., da Cunha M.R., Marques M.M., de Araújo D.R., Marchi J. (2024). Tricalcium phosphate-loaded injectable hydrogel as a promising osteogenic and bactericidal teicoplanin-delivery system for osteomyelitis treatment: An in vitro and in vivo investigation. Biomater. Adv..

[41] Chao S.C., Wang M.-J., Pai N.-S., Yen S.-K. (2015). Preparation and characterization of gelatin–hydroxyapatite composite microspheres for hard tissue repair. Mater. Sci. Eng. C.

[42] Báró M., Sánchez E., Delgado A., Perera A., Evora C. (2002). In vitro-in vivo characterization of gentamicin bone implants. J. Control. Release.

[43] Bhusal P., Rahiri J.L., Sua B., McDonald J.E., Bansal M., Hanning S., Sharma M., Chandramouli K., Harrison J., Procter G. (2018). Comparing human peritoneal fluid and phosphate-buffered saline for drug delivery: Do we need bio-relevant media?. Drug Deliv. Transl. Res..

[44] Hopkins E., Sanvictores T., Sharma S. (2022). Physiology, Acid Base Balance.

[45] Corrigan O.I., Devlin Y., Butler J. (2003). Influence of dissolution medium buffer composition on ketoprofen release from ER products and in vitro–in vivo correlation. Int. J. Pharm..

[46] Ritger P.L., Peppas N.A. (1987). A simple equation for description of solute release I. Fickian and non-fickian release from non-swellable devices in the form of slabs, spheres, cylinders or discs. J. Control. Release.

[47] Bruschi M.L., Bruschi M.L. (2015). 5—Mathematical models of drug release. Strategies to Modify the Drug Release from Pharmaceutical Systems.

[48] Ferraz M.P., Mateus A.Y., Sousa J.C., Monteiro F.J. (2007). Nanohydroxyapatite microspheres as delivery system for antibiotics: Release kinetics, antimicrobial activity, and interaction with osteoblasts. J. Biomed. Mater. Res. Part A.

[49] (2009). Biological Evaluation of Medical Devices—Part 5: Tests for In Vitro Cytotoxicity.

[50] Paul W., Sharma C.P. (1999). Development of Porous Spherical Hydroxyapatite Granules: Application Towards Protein Delivery. J. Mater. Sci. Mater. Med..

[51] Hulbert S.F., Morrison S.J., Klawitter J.J. (1972). Tissue reaction to three ceramics of porous and non-porous structures. J. Biomed. Mater. Res..

[52] Chang B.-S., Lee I.-K., Hong K.-S., Youn H.-J., Ryu H.-S., Chung S.-S., Park K.-W. (2000). Osteoconduction at porous hydroxyapatite with various pore configurations. Biomaterials.

[53] Klose D., Siepmann F., Elkharraz K., Krenzlin S., Siepmann J. (2006). How porosity and size affect the drug release mechanisms from PLGA-based microparticles. Int. J. Pharm..

[54] Sivakumar M., Panduranga Rao K. (2002). Preparation, characterization and in vitro release of gentamicin from coralline hydroxyapatite–gelatin composite microspheres. Biomaterials.

[55] Martins M.A., Santos C., Almeida M.M., Costa M.E.V. (2008). Hydroxyapatite micro- and nanoparticles: Nucleation and growth mechanisms in the presence of citrate species. J. Colloid Interface Sci..

[56] Ashok M., Meenakshi Sundaram N., Narayana Kalkura S. (2003). Crystallization of hydroxyapatite at physiological temperature. Mater. Lett..

[57] Liu D.-M., Yang Q., Troczynski T., Tseng W.J. (2002). Structural evolution of sol–gel-derived hydroxyapatite. Biomaterials.

[58] Raynaud S., Champion E., Bernache-Assollant D., Thomas P. (2002). Calcium phosphate apatites with variable Ca/P atomic ratio I. Synthesis, characterisation and thermal stability of powders. Biomaterials.

[59] Mousia Z., Farhat I.A., Pearson M., Chesters M.A., Mitchell J.R. (2001). FTIR microspectroscopy study of composition fluctuations in extruded amylopectin–gelatin blends. Biopolymers.

[60] Hartgerink J., Beniash E., Stupp S. (2001). Self-Assembly and Mineralization of Peptide-Amphiphile Nanofibers. Science.

[61] Xu Q., Tanaka Y., Czernuszka J.T. (2007). Encapsulation and release of a hydrophobic drug from hydroxyapatite coated liposomes. Biomaterials.

[62] Anderegg T.R., Sader H.S., Fritsche T.R., Ross J.E., Jones R.N. (2005). Trends in linezolid susceptibility patterns: Report from the 2002–2003 worldwide Zyvox Annual Appraisal of Potency and Spectrum (ZAAPS) Program. Int. J. Antimicrob. Agents.

[63] Adali T., Yilmaz E. (2009). Synthesis, characterization and biocompatibility studies on chitosan-graft-poly(EGDMA). Carbohydr. Polym..

